# Encephaloduroarteriosynangiosis for the Treatment of Cerebral Arteriovenous Malformations in the Pediatric Population

**DOI:** 10.7759/cureus.81765

**Published:** 2025-04-05

**Authors:** Matthew Thomas, Dirk Hoening, John Stauffer, Bethany Atkins

**Affiliations:** 1 Pediatrics and Child Health, Lake Erie College of Osteopathic Medicine, Bradenton, USA; 2 General Surgery, Lake Erie College of Osteopathic Medicine, Bradenton, USA; 3 Medicine, Lake Erie College of Osteopathic Medicine, Bradenton, USA; 4 Pediatrics, Baptist Health Jacksonville, Jacksonville, USA

**Keywords:** brain vascular malformation, cerebral avm, encephaloduroarteriosynangiosis (edas), pediatric vascular surgery, spetzler-martin grading

## Abstract

Arteriovenous malformations (AVMs) in the pediatric population are rare but represent a critical cause of spontaneous intracranial hemorrhage. Here we present the case of an 11-year-old boy who developed acute-onset left-sided weakness, slurred speech, and transient hemiparesis. While initial laboratory findings were unremarkable, brain magnetic resonance imaging revealed a large right cerebral AVM. Cerebral angiography confirmed this finding, showing a Spetzler-Martin grade V AVM, supplied by the middle, anterior, and posterior cerebral arteries. The patient was treated using a multimodal approach, including embolization and encephaloduroarteriosynangiosis (EDAS). Postoperatively, he experienced transient worsening of hemiparesis of unknown etiology, which gradually improved. Long-term follow-up demonstrated improved motor function with persistent mild left-sided weakness. This case highlights the significance of multimodal management in high-grade pediatric AVMs. This particular includes the utility of EDAS, originally developed for moyamoya disease, in mitigating ischemia by promoting collateral neovascularization and thus improving neurological symptoms.

## Introduction

In a normal vascular network, oxygenated blood travels through the arterial system into a capillary bed, through which it supplies an organ. The blood is drained through the venous system and returns to the heart. In the case of an arteriovenous malformation (AVM), blood is directly shuttled through a fistula from an artery directly into a vein, likely due to failure of primitive arteriovenous shunts to undergo apoptosis during embryogenesis [1]. 

The resulting absence of a capillary bed connecting the two vascular networks creates a high-flow shunt that prevents oxygenated blood from properly supplying the organ in which the AVM resides. This can cause a number of problems depending on the organ system affected by an AVM, with the central nervous system being by far the most common [1]. Central nervous system AVMs can lead to headaches, seizures, transient neurologic deficits, and clinical signs of venous hypertension such as pulsatile tinnitus [2]. However, the most common and serious manifestation of AVMs is intracerebral hemorrhage, with the annual risk of bleeding from an AVM ranging between 0.9% and 4% [3].

Arteriovenous malformations typically present in adulthood, as only 3% of AVMs are represented among the pediatric population. However, they are the most common cause of spontaneous intraparenchymal hemorrhage in children [4]. Thus, despite their relative rarity, they should be a subject of intensive study in order to increase clinical awareness of these vascular malformations among pediatricians. By highlighting this multimodal management approach, including the use of indirect revascularization surgery known as the encephaloduroarteriosynangiosis (EDAS) procedure, this study aims to improve early detection and treatment strategies for better outcomes in this unique patient population. Here, we highlight such a case of a high-grade AVM in a child and review the existing literature regarding the management of pediatric AVMs.

## Case presentation

We present an 11-year-old boy with a history of attention-deficit/hyperactivity disorder (ADHD), auditory processing disorders, and “memory problems” brought to the emergency department (ED) by their mother for acute-onset left-sided weakness. In addition to the sudden-onset weakness, the patient began drooling water during attempted hydration. The patient also developed slurred speech and fell to the ground. 

Upon further review of his medical history, the mother reported one prior episode of loss of consciousness three months ago when he was fishing, which was attributed to the heat exposure. Since then, he had experienced multiple episodes, which were typically in settings where he was exposed to the heat. They were immediately brought to the ED, by which time he had largely experienced a spontaneous resolution of symptoms.

**Figure 1:**
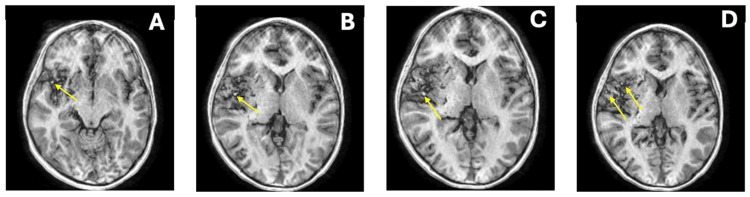
Axial T1-weighted MRI images (A-D) demonstrating a large vascular malformation in the right cerebral hemisphere (yellow arrows), with associated mass effect and partially empty sella

**Figure 2:**
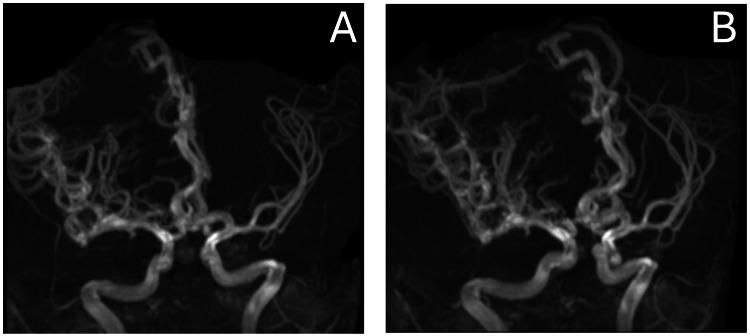
3D time-of-flight magnetic resonance angiography (MRA) images (A-B) illustrating an extensive arteriovenous malformation (AVM) affecting the right cerebral hemisphere. The arterial feeders (from the right anterior cerebral artery, right middle cerebral artery, and right posterior cerebral artery) and deep venous drainage (vein of Galen, enlarged right vein of Labbe, and superior sagittal sinus) are clearly visualized. Annotations indicate key vessels involved.

During workup in the ED, complete blood count, partial thromboplastin time (PTT), prothrombin time (PT), and comprehensive metabolic panel (CMP) were within normal limits (Tables 1-3). The magnetic resonance imaging (MRI) revealed a large vascular malformation likely representing an AVM involving a large section of the right cerebral hemisphere as well as a partially empty sella (Figure 1). Further analysis via angiogram revealed a right-sided AVM supplied by the middle cerebral artery, anterior cerebral artery, and posterior cerebral arteries, measuring greater than 6 cm with superficial and deep venous drainage without any evidence of associated internal aneurysm, Spetzler-Martin grade V (Figure 2) [5]. Angiogram also revealed fenestration of the right vertebral artery, V4 segment. Additionally, pituitary labs were obtained per endocrinology recommendation and further revealed a low cortisol level (8.3 mcg/dL) due to secondary adrenal insufficiency after a low-dose adrenocorticotropic hormone (ACTH) stimulation test (reference range: cortisol > 12.6 mcg/dL). Endocrinology consult suggested a maintenance dose of cortisol with stress dosing as needed. Neurology consult suggested a therapeutic dose of levetiracetam to prevent seizures and highlighted the need for future corrective surgery.

**Table 1:** Complete blood count with differential

Results name	Values/Results	Units	Reference value
Eosinophils - absolute	0.2	K/mcL	0.00-0.45
Monocytes - absolute	0.64	K/mcL	0.20-0.90
Red blood cell count	4.91	mil/mcL	3.90-5.30
Basophils - absolute	0.04	K/mcL	0.00-0.10
Basophils %	1	%	0-2
Hematocrit	42.9	%	35.0-45.0
Mean corpuscular hemoglobin concentration (MCHC)	33.4	g/dL	30.0-37.0
Neutrophils - absolute	2.82	K/mcL	1.80-8.00
Platelet count	285	K/mcL	150-450
Mean corpuscular hemoglobin (MCH)	29.2	pg	25.0-35.0
Monocytes %	10	%	3-15
MPV	6.9	fL	7.0-11.0
Lymphocytes - absolute	2.89	K/mcL	1.00-5.00
Eosinophils %	3	%	0-7

**Table 2:** Results of the coagulation studies

Results Name	Values/Results	Units	Reference value
Prothrombin time (PT)	11.8	Seconds	11.8-15.0
Partial thromboplastin time (PTT)	27.5	Seconds	22.9-37.8
International normalized ratio (INR)	0.9		

**Table 3:** Comprehensive metabolic panel

Result Name	Values/Results	Units	Reference value
Chloride	100	mEg/L	98-110
Calcium	9.5	mg/dL	8.5-10.1
Carbon dioxide	26	mEq/L	22-32
Creatinine	0.47	mg/dL	0.40-1.40
Sodium	141	mEg/L	135-145
Glucose level	104	mg/dL	70-100
Albumin	4.9	g/dL	3.4-5.1
Blood urea nitrogen	7	mg/dL	7-23
Potassium	3.7	mEg/L	3.2-5.1
Protein	7.7	g/dL	5.7-8.2

The patient was admitted two months later for a staged surgery for his right cerebral proliferative angiopathy. He underwent endovascular embolization of the right frontal superficial temporal artery (STA) branch with interventional radiology the same day without complications. The following day, the patient went to the operating room for the EDAS procedure and brain specimen collection. All neuromonitoring remained at baseline throughout the case, and the patient returned to the intensive care unit in stable condition.

The following day, on postoperative day one (POD 1), the patient experienced weakness of his left upper extremity and left foot above his baseline presentation of left hemibody weakness. Thus, a computed tomography (CT) head was ordered on POD 2 for evaluation of possible subdural or epidural bleed, which was unremarkable. His weakness resolved on POD 3; he tolerated an oral diet and had a return of bowel and urine function upon discharge. He was discharged home in stable condition with his mother.

The patient’s secondary adrenal insufficiency due to the empty sella turcica was later stabilized with 10 mg hydrocortisone in the morning and 7.5 mg in the evening. The patient had residual mild hemiparesis of the left non-dominant side of unspecified etiology but has improved in strength and mobility since the procedure. Additionally, the patient has poor use of the left hand but can use the whole hand or elbow to assist and has otherwise learned compensatory behavior. He continues to walk community distances but does frequently trip. Otherwise, the patient remains in good health, with no significant neurologic defect, and plans to continue postgraduate education.

## Discussion

It is crucial to follow the development of AVMs as they are not static. As time progresses, malformations can become larger due to increased blood flow to the area, and this enlargement is the biggest predictor of morbidity [1]. In this case, the patient’s symptoms worsened when exposed to heat due to the blood shunt speeding up through the AVM. In a pediatric case like this one, monitoring the AVM every six to 12 months is recommended. 

The most widely accepted scale for classifying AVMs is the Spetzler-Martin scale, first proposed in 1986 by two neurosurgeons, Dr. Robert Spetzler and Dr. Neil Martin. This grading scale classifies AVMs into six categories, grades I-V, based on size, venous drainage (superficial vs. deep), and the neurological eloquence of adjacent brain tissue. They also created a special designation of grade VI for AVMs considered to be ‘essentially inoperable’ [5]. As per the Spetzler-Martin grading, an AVM is categorized as small (<3 cm) if it is scored as one point, medium (3-6 cm) if it is scored as two points, or large (>6 cm) if it is scored as three points. Venous drainage has two classifications, which are superficial (0 points) or deep (one point). The eloquence of the surrounding brain tissue is also taken into account, with non-eloquent areas scoring 0 points and eloquent areas scoring one point. The total grade is determined by adding these scores together, with grade VI specifically designated for AVMs that are considered 'essentially inoperable' [5].

There are several ways to treat AVMs depending on the Spetzler-Martin grade [6]. Surgical resection remains the gold standard due to having the lowest recurrence rate and has become a highly effective method of treatment with advances in microsurgical technology [7]. Other commonly employed tactics include radiosurgery and embolization, often used in conjunction with surgical resection in order to achieve complete obliteration of the lesion. Embolization has a higher rate of recurrence when used as solo therapy than resection, which is why it is primarily used in conjunction with surgical resection [8]. Conservative management and clinical observation are also considered in situations where the risks of therapy outweigh the benefits [4]. In this case, the EDAS procedure was performed. The EDAS procedure is an indirect revascularization surgery where the STA is dissected out while leaving it connected to its blood source and then placed on top of the brain tissue [9]. This new blood flow within the brain will promote collateral blood flow over time, perfusing the ischemic tissue [10]. The neovascularization resulting from the EDAS procedure improved this patient's strength and mobility over time, demonstrating the significant enhancement in quality of life that this treatment can provide.

The EDAS procedure was first performed on patients with moyamoya disease, which is a condition where blood vessels in the brain gradually become stenosed [9]. This condition presents similarly to AVMs with ischemic strokes, transient ischemic attacks, seizures, weakness or hemiparesis, and intracerebral hemorrhages [11]. New blood vessels will grow in response to the brain ischemia to perfuse the brain, but these newly formed vessels are fragile and prone to hemorrhaging, which is the most severe complication [10]. After finding success in reducing the number of strokes in Moyamoya disease patients, EDAS was performed in other brain ischemia diseases like AVMs with success in reducing strokes and hemorrhages [12]. However, the EDAS procedure may carry its own risks, which can be displayed by this patient's transient weakness. While the specific etiology of this weakness was not determined, one possible cause may be a well-documented complication known as cerebral hyperperfusion syndrome (CHS), which is recognized in 6.7% to 38.2% of cases following cerebral revascularization. Cerebral hyperperfusion syndrome frequently presents with headaches, seizures, language impairment, and disturbances in motor or sensory function [13]. While the specific cause of CHS is unknown, it is a serious condition that may cause fatal hemorrhage in a small subsection of the population. Further research suggests that maintaining tightly regulated blood pressure and ensuring adequate hydration are crucial in managing postoperative CHS, as they help mitigate the risk of hemodynamic shifts [13].

## Conclusions

This case highlights the complexity and importance of managing high-grade pediatric AVMs, particularly through innovative surgical techniques like EDAS, radiosurgery, and embolization. While AVMs in children are rare occurrences, they pose significant risks of intracranial ischemia and hemorrhage, necessitating vigilant monitoring and individualized treatment strategies. The use of EDAS in this patient successfully promoted alternative neovascularization, reducing ischemic risk and improving neurologic function in this patient. This underscores the potential for EDAS to be adapted for other ischemic conditions, improving quality of life and functional outcomes in affected patients. Given the unique challenges these malformations present in children, more research should be done in order to study the progression, management, and long-term effects of AVMs.

## References

[1] Greene AK, Orbach DB (2011). Management of arteriovenous malformations. Clin Plast Surg.

[2] Barreau X, Marnat G, Gariel F, Dousset V (2014). Intracranial arteriovenous malformations. Diagn Interv Imaging.

[3] Murthy SB, Merkler AE, Omran SS (2017). Outcomes after intracerebral hemorrhage from arteriovenous malformations. Neurology.

[4] El-Ghanem M, Kass-Hout T, Kass-Hout O (2016). Arteriovenous malformations in the pediatric population: review of the existing literature. Interv Neurol.

[5] Spetzler RF, Martin NA (1986). A proposed grading system for arteriovenous malformations. J Neurosurg.

[6] Mao A, Khuddus N, Duong HD (2022). Pediatric cerebral Spetzler-Martin grade 5 arteriovenous malformation. Cureus.

[7] Shaligram SS, Winkler E, Cooke D, Su H (2019). Risk factors for hemorrhage of brain arteriovenous malformation. CNS Neurosci Ther.

[8] Raymond J, Gentric JC, Magro E (2023). Endovascular treatment of brain arteriovenous malformations: clinical outcomes of patients included in the registry of a pragmatic randomized trial. J Neurosurg.

[9] Alamri A, Hever P, Cheserem J, Gradil C, Bassi S, Tolias CM (2019). Encephaloduroateriosynangiosis (EDAS) in the management of moyamoya syndrome in children with sickle cell disease. Br J Neurosurg.

[10] Kono K, Terada T (2014). Encephaloduroarteriosynangiosis for cerebral proliferative angiopathy with cerebral ischemia. J Neurosurg.

[11] Scott RM, Smith ER (2009). Moyamoya disease and moyamoya syndrome. N Engl J Med.

[12] Chan JL, Quintero-Consuegra MD, Babadjouni RM (2022). Encephaloduroarteriosynangiosis operative technique and intraoperative anesthesia management: treatment from both sides of the curtain. Oper Neurosurg (Hagerstown).

[13] Shi Z, Wu L, Wang Y, Zhang H, Yang Y, Hang C (2023). Risk factors of postoperative cerebral hyperperfusion syndrome and its relationship with clinical prognosis in adult patients with moyamoya disease. Chin Neurosurg J.

